# Antiarrhythmic drug therapy after catheter ablation for atrial fibrillation—Insights from the German Ablation Registry

**DOI:** 10.1002/prp2.880

**Published:** 2021-10-19

**Authors:** Ruben Schleberger, Andreas Metzner, Karl‐Heinz Kuck, Dietrich Andresen, Stephan Willems, Ellen Hoffmann, Thomas Deneke, Lars Eckardt, Johannes Brachmann, Matthias Hochadel, Jochen Senges, Andreas Rillig

**Affiliations:** ^1^ Department of Cardiology University Heart and Vascular Center University Medical Center Hamburg‐Eppendorf Hamburg Germany; ^2^ LANS Cardio Hamburg Germany; ^3^ Department of Cardiology Protestant Hospital Hubertus Berlin Germany; ^4^ Department of Cardiology Asklepios Clinic St. Georg Hamburg Germany; ^5^ Department of Cardiology and Internal Intensive Care Medicine Heart Center Munich‐Bogenhausen Bogenhausen Hospital Munich Germany; ^6^ Department of Cardiology Rhön Clinic Campus Bad Neustadt Bad Neustadt a. d. Saale Germany; ^7^ Department of Cardiology (Electrophysiology) University Hospital Münster Münster Germany; ^8^ Department of Cardiology, Angiology and Pneumology Coburg Hospital Coburg Germany; ^9^ Stiftung für Herzinfarktforschung (IHF) Ludwigshafen Germany

**Keywords:** antiarrhythmic drug therapy, atrial fibrillation, catheter ablation, outcome

## Abstract

Data on the optimal treatment strategy for antiarrhythmic drug therapy (AAD) after catheter ablation for atrial fibrillation (AF) are inconsistent. The present study investigates whether postinterventional AAD leads to an improved long‐term outcome. Patients from the prospective German Ablation Registry (*n* = 3275) discharged with or without AAD after catheter ablation for AF were compared regarding the rates of recurrences, reablations and cardiovascular events as well as patient reported outcomes during 12 months follow‐up. In patients with paroxysmal AF (*n* = 2138) the recurrence rate did not differ when discharged with (*n* = 1051) or without (*n* = 1087) AAD (adjusted odds ratio (OR) 1.13, 95% confidence interval (CI) [0.95–1.35]). The reablation rate was higher and reduced treatment satisfaction was reported more often in those discharged with AAD (reablation: OR 1.30, 95% CI [1.05–1.61]; reduced treatment satisfaction: OR 1.76, 95% CI [1.20–2.58]). Similar rates of recurrences, reablations and treatment satisfaction were found in patients with persistent AF (*n* = 1137) discharged with (*n* = 641) or without (*n* = 496) AAD (recurrence: OR 1.22, 95% CI [0.95–1.56]; reablation: OR 1.21, 95% CI [0.91–1.61]; treatment satisfaction: OR 1.24, 95% CI [0.74–2.08]). The incidence of cardiovascular events and mortality did not differ at follow‐up in patients discharged with or without AAD.

In conclusion, the rates of recurrences, cardiovascular events and mortality did not differ between patients discharged with or without AAD after AF catheter ablation. However, AAD should be considered carefully in patients with paroxysmal AF, in whom it was associated with a higher reablation rate and reduced treatment satisfaction.

**Clinical trial registration:** The trial has been registered under the number NCT01197638.

## Graphical abstract

Prescription of antiarrhythmic drug therapy after catheter ablation for atrial fibrillation is a common daily clinical practice, but there is conflicting evidence on its benefits. In our analysis from the German Ablation Registry the rates of recurrences, cardiovascular events and mortality did not differ between patients discharged with or without antiarrhythmic drug therapy after catheter ablation. However, antiarrhythmic drug therapy should be considered carefully in patients with paroxysmal atrial fibrillation, in whom it was associated with a higher reablation rate and reduced treatment satisfaction.

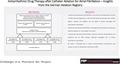

**Abbreviations**
AAD: antiarrhythmic drug therapy
AF: atrial fibrillation
CFAE: complex fractionated atrial electrograms
CI: confidence interval
HR: hazard ratio
OR: odds ratio
PVI: pulmonary vein isolation

## 1 INTRODUCTION

Recurrences of atrial fibrillation (AF) after catheter ablation are frustrating for patients and physicians as they go along with a reduced quality of life and an increased number of rehospitalizations.^1^ In addition to electrical pulmonary vein reconnection,^2^ a pathophysiological explanation for recurrences might be an early inflammatory reaction of the left atrial perimyocardial structures in response to the ablation procedure, leading to a temporary increase in atrial stretch and pressure.^3^ Previous studies have shown that arrhythmia recurrences during the first year after ablation may be a predictor for a worse long‐term outcome.^4^ A combined interventional and long‐term drug‐based antiarrhythmic therapy might have the potential to further reduce the AF burden.^5^, ^6^ The evidence regarding postinterventional antiarrhythmic drug therapy (AAD) after catheter ablation is yet sparse and incongruent, leaving clinicians without a clear recommendation.^7^, ^8^, ^9^

Our present study's aim was to analyze the effect of prescription of AAD at discharge after AF catheter ablation on the recurrence and reablation rate, mortality and patient reported outcomes during a 12‐month follow‐up period.

## 2 MATERIALS AND METHODS

### 2.1 German Ablation Registry

The German Ablation Registry (NCT01197638) is a prospective multicenter registry that is managed by the “Institut für Herzinfarktforschung” (IHF). Patients >18 years of age were enrolled in 55 participating centers in Germany between January 2007 and January 2010. Trial development, data acquisition, and clinical monitoring were organized by the IHF.

### 2.2 Patient selection and study design

**FIGURE 1:**
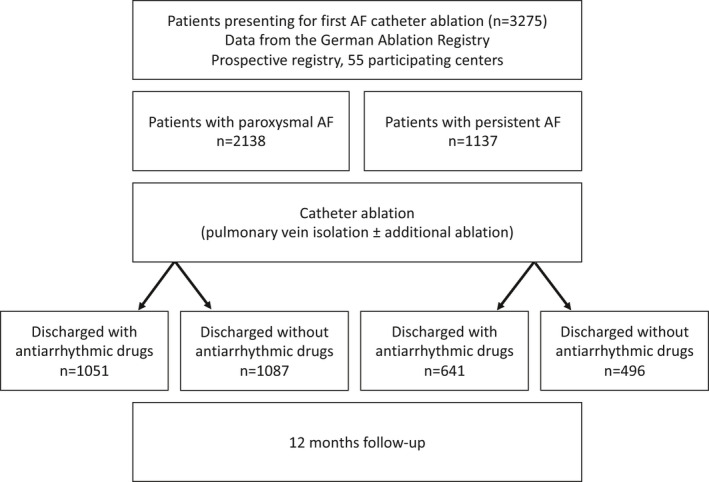
Flow‐chart of the study design. Patients from the German Ablation Registry presenting for their first catheter ablation for atrial fibrillation were included into the analysis. Patients with paroxysmal or persistent atrial fibrillation were analyzed separately. The long‐term outcome of individuals taking antiarrhythmic drug therapy of Vaughan Williams class I or class III at discharge after ablation was compared to the outcome of individuals without prescription of specific antiarrhythmic drugs. Abbreviations: AF, atrial fibrillation

Patients from the German Ablation Registry presenting for their first AF catheter ablation were included into the analysis (see Figure 1). The long‐term outcome of individuals with prescription AAD of Vaughan Williams class I or class III (AAD group) at the timepoint of discharge after ablation was compared to the outcome of individuals without prescription of specific antiarrhythmic drugs (no AAD group). Betablockers were accepted as a baseline therapy in both groups. Patients with AV‐node ablation, arrhythmia recurrence before discharge or patients taking class IV AAD or a combination of class I/class III AAD were excluded from analysis.

### 2.3 Ablation procedure

Patients were treated according to the respective institutional standards and the contemporary guidelines as described before.^9^ In brief, patients underwent the procedure in the fasting state under sedation using midazolam or propofol and fentanyl or sufentanil in most cases. Oral anticoagulation with vitamin K antagonists was stopped a few days before the ablation procedure and substituted with low‐molecular heparin. During procedures intravenous heparin was administered aiming at an activated clotting time of 250–300 s. An electroanatomical 3D‐mapping system such as Carto^®^ (Biosense Webster) or EnSite NavX™ (St. Jude) and the energy source (radiofrequency or cryothermal energy) was used at the operator's discretion.

### 2.4 Analysis of follow‐up data

In addition to the clinical follow‐up which was performed according to local standards of the respective participating institution, a standardized patient telephone interview was performed 12 months post ablation by the IHF. The patients answered a questionnaire (see Supplement) including information on arrhythmia recurrences, repeat ablations, adverse events, and quality of life. The patients’ subjective perception of the ablation therapy was analyzed and classified as successful, partly successful, or unsuccessful. Recurrences were defined as any episode of atrial fibrillation or atrial tachycardia lasting for at least 30 s. Possible arrhythmia recurrences were counted if evidence by ECG documentation or medical treatment was reported. A selection of adverse events (see details in Table S1) was reported on the standardized questionnaire and classified as moderate or severe. The endpoints mortality, MACE (major adverse cardiac event; including death, myocardial infarction), MACCE (major adverse cardiac and cerebrovascular event; including death, myocardial infarction, ischemic stroke) and a quadruple safety endpoint (death, myocardial infarction, ischemic stroke, major bleeding) were analyzed separately.

### 2.5 Statistics

Descriptive statistics are presented as count and percentage for categorical variables and as mean ± standard deviation or median (interquartile range) in case of skewed data for continuous variables. Patient characteristics were compared using Pearson chi‐square tests or Mann–Whitney *U* tests. For infrequent hospital complications Fisher's exact test was applied where indicated in the tables. Mortality, MACE, MACCE and the quadruple safety endpoint were analyzed as time‐to‐event data using the Kaplan–Meier method and log rank test to estimate and compare the 12‐months event rates, and Cox regression to calculate hazard ratios (HR) with 95% confidence intervals (CI). Other follow‐up outcomes were recorded as binary data from patients surviving at the time of follow‐up and analyzed by multivariable logistic regression models with results presented as odds ratios (OR). In order to adjust for baseline imbalances, the regression models for different outcome parameters included the factors age, sex, coronary artery disease, left ventricular ejection fraction, ablation of complex fractionated atrial electrograms (CFAE), linear lesions, cryo ablation and procedural success, as well as long‐standing persAF in the group with persistent AF. The reported *p*‐values are two‐sided; *p*‐values less than 0.05 are considered statistically significant. Statistical computations were performed using the statistical software SAS version 9.4 (SAS Institute Inc.).

## 3 RESULTS

### 3.1 Baseline parameters

In total, 3275 patients with AF were included into the study. Baseline parameters are shown in Table 1 for patients with paroxysmal AF (pAF, *n* = 2138) and in Table 2 for patients with persistent AF (persAF, *n* = 1137).

**TABLE 1:** Baseline parameters—patients with paroxysmal atrial fibrillation

	AAD (*n* = 1051)	No AAD (*n* = 1087)	*p*‐Value
Male	62.1%	65.2%	.14
Age, years	59.9 ± 10.4	59.9 ± 11.0	.65
Cardiac disease	28.5%	35.0%	.001
Ischemic cardiomyopathy	12.8%	20.5%	
Dilative cardiomyopathy	2.2%	2.0%	
Hypertrophic cardiomyopathy	0.8%	0.7%	
Vitium	5.8%	5.8%	
LV‐Function (*n* = 984/986)			.099
>50%	91.1%	88.7%	
41%–50%	6.4%	8.9%	
31%–40%	2.0%	1.7%	
≤30%	0.5%	0.6%	
Diabetes	7.9%	7.6%	.82
Chronic kidney disease^a^	3.3%	2.0%	.41
Arterial hypertension^a^	63.4%	55.3%	.11
COPD^a^	1.1%	1.0%	.91
Peripheral artery disease^a^	0.6%	1.0%	.63
Pacemaker/ICD	6.8%	5.7%	.31
CHA_2_DS_2_‐VASc score^a^	1.8 ± 1.3	1.6 ± 1.3	.18

Note

Values are presented as percent (%) of available data sets or mean ± standard deviation. *p* < .05 is considered statistically significant.

Abbreviations: AAD, antiarrhythmic drugs; COPD, chronic obstructive pulmonary disease; ICD, implanted cardioverter‐defibrillator; ICM, ischemic cardiomyopathy; LV, left‐ventricular. Antiarrhythmic drugs include class I/III agents.

^a^ Data available for 12%–17% of patients due to later inclusion of the variable into the study.

**TABLE 2:** Baseline parameters—patients with persistent atrial fibrillation

	AAD (*n* = 641)	No AAD (*n* = 496)	*p*‐Value
Male	74.9%	75.4%	.84
Age, years	61.0 ± 9.9	62.0 ± 10.2	.090
Cardiac disease	41.2%	51.0%	.001
Ischemic cardiomyopathy	18.1%	25.2%	
Dilative cardiomyopathy	5.3%	6.0%	
Hypertrophic cardiomyopathy	1.0%	1.8%	
Vitium	10.0%	8.7%	
LV‐Function (*n* = 592/448)			.001
>50%	81.4%	72.5%	
41%–50%	11.8%	19.2%	
30%–40%	4.9%	6.0%	
<30%	1.9%	2.2%	
Diabetes	5.6%	10.7%	.002
Chronic kidney disease^a^	3.5%	1.6%	.45
Arterial hypertension^a^	71.1%	54.7%	.028
COPD^a^	1.8%	4.7%	.27
Peripheral artery disease^a^	2.7%	0.0%	.19
Pacemaker/ICD	4.7%	6.7%	.15
CHA_2_DS_2_‐VASc score^a^	1.9+−1.3	1.7+−1.2	.48

Note

Values are presented as percent (%) of available data sets or mean ± standard deviation. *p* < .05 is considered statistically significant.

Abbreviations: AAD, antiarrhythmic drugs; COPD, chronic obstructive pulmonary disease; ICD, implanted cardioverter‐defibrillator; ICM, ischemic cardiomyopathy; LV, left‐ventricular. Antiarrhythmic drugs include class I/III agents.

^a^ Data available for 12%–17% of patients due to later inclusion of the variable into the study.

### 3.2 Procedural characteristics

Pulmonary vein isolation (PVI) was conducted in all patients. Linear lesions were added in 13.5% (142/1051) of pAF patients discharged with AAD and 10.3% (112/1086) discharged without AAD (*p* = .022, see Table 3). CFAE were ablated in 3.1% (29/931) versus 1.6% (16/982, *p* = .032).

**TABLE 3:** Procedural parameters—patients with paroxysmal atrial fibrillation

	AAD (*n* = 1051)	No AAD (*n* = 1087)	*p*‐Value
Energy source (%)			
Radiofrequency	74.7%	69.7%	.011
Cryo	23.5%	29.3%	.003
Other	1.8%	1.0%	.12
Additional linear lesion	13.5%	10.3%	.022
Thereof cavotricuspid isthmus	51.4%	66.1%	.019
Ablation of CFAE (*n* = 931/982)	3.1%	1.6%	.032
Radiofrequency duration, seconds (*n* = 641/539)	2344 (1440; 3060)	2700 (1920; 3540)	.001
Fluoroscopy duration, minutes (*n* = 974/955)	31.4 ± 20.1	35.8 ± 24.0	.001
Area dose product, cGy*cm^2^	3470 (1631; 6996)	3685 (1900; 7357)	.10
Procedure duration, minutes	173.3 ± 72.3	176.2 ± 67.4	.19
Acute success	97.1%	96.6%	.46

Note

Values are presented as percent (%) of available data sets, median (interquartile range) or mean ± standard deviation. *p* < .05 is considered statistically significant.

Abbreviations: AAD, antiarrhythmic drugs; CFAE, complex fractionated atrial electrograms.

In patients with persAF, additional linear lesions besides PVI were ablated in 22.9% (147/641) of patients of the AAD group and 17.7% (88/496) of patients in the no AAD group (*p* = .032, see Table 4). Furthermore, CFAE were ablated in 22.9% (129/563) versus 17.2% (77/477, *p* = .026).

**TABLE 4:** Procedural parameters—patients with persistent atrial fibrillation

	AAD (*n* = 641)	No AAD (*n* = 496)	*p*‐Value
Energy source (%)			
Radiofrequency	88.1%	90.3%	.24
Cryo	11.4%	8.7%	.13
Other	0.5%	1.0%	.28
Additional linear lesion	22.9%	17.7%	.032
Thereof cavotricuspid isthmus	45.6%	54.5%	.18
Ablation of CFAE (*n* = 563/447)	22.9%	17.2%	.026
Radiofrequency duration, seconds (*n* = 372/204)	3000 (1982; 4588)	3080 (1980; 4826)	.58
Fluoroscopy duration, minutes (*n* = 582/435)	37.3 ± 27.7	43.6 ± 28.0	.001
Area dose product, cGy*cm^2^ (*n* = 610/455)	3900 (2213; 6989)	4500 (2469; 7593)	.10
Procedure duration, minutes	194.8 ± 72.5	189.3 ± 66.6	.36
Acute success	96.7%	93.3%	.008

Note

Values are presented as percent (%) of available data sets, median (interquartile range) or mean ± standard deviation. *p* < .05 is considered statistically significant.

Abbreviations: AAD, antiarrhythmic drugs; CFAE, complex fractionated atrial electrograms.

### 3.3 Long‐term outcome

Follow‐up data were available for 97.8% (3203/3275) of patients. The mean follow‐up duration was 477 ± 106 days.

**FIGURE 2:**
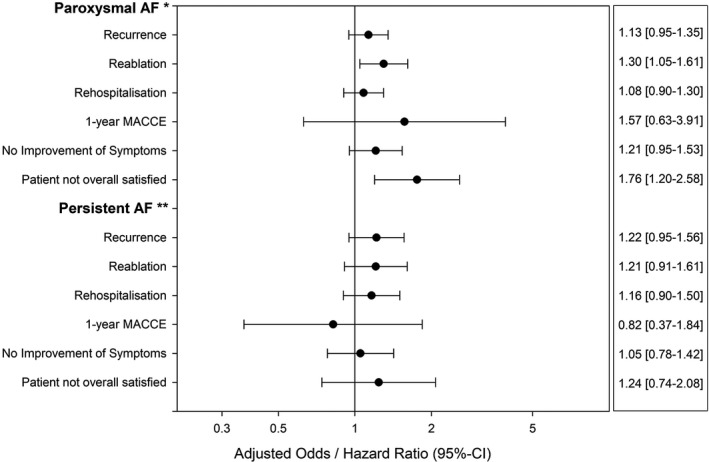
Adjusted outcome analysis of outcome after catheter ablation for atrial fibrillation. The 12‐months outcome of patients with postinterventional antiarrhythmic drug therapy was compared to patients without postinterventional antiarrhythmic drug therapy. Antiarrhythmic drugs comprised Vaughan Williams Class I and III. Betablocker therapy was allowed in both groups. The upper part of the figure shows patients with paroxysmal atrial fibrillation, the lower part shows patients with persistent atrial fibrillation. The values left of the middle axis stands for a benefit of antiarrhythmic drug therapy. Values right of the axis stand for a disadvantage of antiarrhythmic drug therapy. Adjusted odds ratio or hazard ratio with 95% confidence interval are shown. *Adjusted for age, sex, coronary artery disease, left ventricular ejection fraction>50%, ablation of complex fractionated atrial electrogram, linear lesions, cryo‐ablation, acute procedural success. **Adjusted for above mentioned factors (*) plus long‐standing persistent atrial fibrillation in addition. Abbreviations: AF, atrial fibrillation; CI, confidence interval; MACCE, major adverse cardiac and cerebrovascular event

After pAF catheter ablation, recurrences occurred in 43.5% (438/1008) of patients discharged with AAD versus 40.2% (421/1046) of patients discharged without AAD. The multivariable regression analysis (see Figure 2) showed no significant difference regarding recurrences between groups: OR 1.13, 95% CI [0.95–1.35]. Reablations were more often performed in patients that were discharged with AAD (OR 1.30, 95% CI [1.05–1.61]). Hospitalizations during follow‐up were similar in both groups: OR 1.08, 95% CI [0.90–1.30].

At the end of the follow‐up period the use of Class I/III AAD had decreased from 100% to 42.0% (405/965) in the AAD group and increased from 0% to 16.1% (161/1005) in the no AAD group.

After persAF catheter ablation, recurrences were diagnosed in 48.0% (293/611) of patients in the AAD group and in 41.9% (197/470) of patients discharged without AAD. The multivariable regression analysis (see Figure 2) revealed no significant difference regarding recurrences (OR 1.22, 95% CI [0.95–1.56]), reablations (OR 1.21, 95% CI [0.91–1.61]) and rehospitalizations (OR 1.16, 95% CI [0.90–1.50], see Figure 2).

Patients discharged with AAD were still on medication at last follow‐up in 40.7% (234/547) of cases while 20.5% (86/448) of patients that were initially discharged without AAD were on medication at last follow‐up.

### 3.4 Adverse events during follow‐up

Patients with pAF experienced severe adverse events during follow‐up in 1.5% (15/969, AAD group) versus 1.2% (12/1010, no AAD group, *p* = .49) and moderate adverse events in 9.1% (73/803, AAD group) versus 7.4% (66/888, no AAD group, *p* = .22). The compound endpoint consisting of death, myocardial infarction, and stroke (MACCE) occurred in 0.6% of patients discharged with AAD and 0.6% of patients discharged without AAD (Kaplan–Meier estimates, *p* = .95; adjusted HR 1.57, 95% CI [0.63–3.91]). Five patients treated for pAF died during the follow‐up period in both groups, respectively (see Table 5).

**TABLE 5:** 12‐Months clinical outcome—patients with paroxysmal atrial fibrillation

	AAD (*n* = 1051)	No AAD (*n* = 1087)	*p*‐Value
Follow‐up available	1027 (97.7%)	1063 (97.8%)	.91
Follow‐up duration, days	474.8 ± 91.7	467.0 ± 91.9	.009
Adverse events during follow‐up			
Severe adverse event	1.5%	1.2%	.49
Moderate adverse event	9.1%	7.4%	.22
Mortality^a^	0.2%	0.4%	.44
MACE (death, MI)^a^	0.3%	0.4%	.74
MACCE (death, MI, stroke)^a^	0.6%	0.6%	.95
Quadruple safety endpoint (death, MI, stroke, major bleeding)^a^	1.4%	1.3%	.92
Recurrence during follow‐up	43.5%	40.2%	.14
Reablation during follow‐up	23.1%	18.8%	.017
Hospitalization during follow‐up	44.5%	43.1%	.51
AAD at end of follow‐up			
AAD Class I	27.5%	8.1%	.001
AAD Class II	67.0%	72.1%	.014
AAD Class III	14.5%	8.0%	.001
AAD Class IV	2.0%	1.9%	.90

Note

Values are presented as percent (%) of available data sets or mean ± standard deviation. *p* < .05 is considered statistically significant.

Abbreviations: AAD, antiarrhythmic drugs; MACCE, major adverse cardiac and cerebrovascular event; MACE, major adverse cardiac event; MI, myocardial infarction. Details on the types of adverse events are shown in Table S1b.

^a^ Kaplan–Meier estimates at 366 days after index discharge, compared by log‐rank test.

Likewise, there were no significant differences for persAF patients (severe adverse events: AAD group: 2.1% (12/582) versus no AAD group: 2.7% (12/449), *p* = .52; moderate adverse events: AAD group: 6.6% (32/484) versus no AAD group: 8.2% (32/391), *p* = .37. MACCE occurred in 1.9% versus 2.3% of patients (Kaplan–Meier estimates, *p* = .68; adjusted HR 0.82, 95% CI [0.37–1.84]). Death during follow‐up occurred in 1.1% in the AAD group versus 1.2% in the no AAD group (*p* = .85, see Table 6).

**TABLE 6:** 12‐months clinical outcome—patients with persistent atrial fibrillation

	AAD (*n* = 641)	No AAD (*n* = 496)	*p*‐Value
Follow‐up available	627 (97.8%)	486 (98.0%)	.85
Follow‐up duration, days	492.4 ± 163.8	482.1 ± 95.7	.36
Adverse events during follow‐up			
Severe adverse event	2.1%	2.7%	.52
Moderate adverse event	6.6%	8.2%	.37
Mortality^a^	1.1%	1.2%	.85
MACE (death, MI)^a^	1.3%	1.4%	.81
MACCE (death, MI, stroke)^a^	1.9%	2.3%	.68
Quadruple safety endpoint (death, MI, stroke, major bleeding)^a^	2.9%	3.3%	.68
Recurrence during follow‐up	48.0%	41.9%	.048
Reablation during follow‐up	27.2%	23.8%	.21
Hospitalization during follow‐up	51.0%	48.7%	.45
AAD at end of follow‐up			
AAD Class I	15.5%	6.9%	.001
AAD Class II	66.2%	72.5%	.030
AAD Class III	25.3%	12.3%	.001
AAD Class IV	1.9%	2.7%	.41

Note

Values are presented as percent (%) of available data sets or mean ± standard deviation. *p* < .05 is considered statistically significant.

Abbreviations: AAD, antiarrhythmic drugs; MACCE, major adverse cardiac and cerebrovascular event; MACE, major adverse cardiac event; MI, myocardial infarction. Details on the types of adverse events are shown in Table S1b.

^a^ Kaplan–Meier estimates at 366 days after index discharge, compared by log‐rank test.

Further details for all groups are shown in Table S1.

### 3.5 Quality of life

#### 3.5.1 Symptom status at follow‐up

**FIGURE 3:**
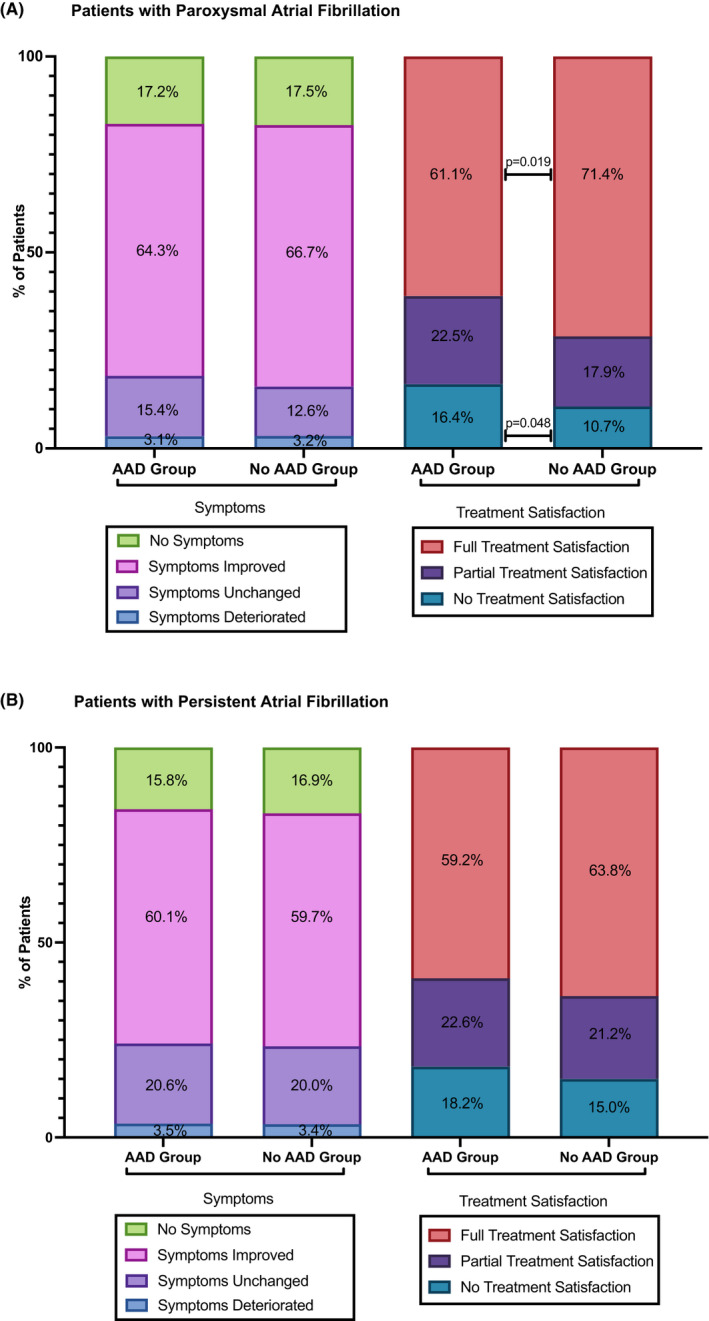
12‐Months outcome analysis of patient reported outcome (Univariate Analysis). (A) Patients with paroxysmal atrial fibrillation. (B) Patients with persistent atrial fibrillation. The patient reported outcome parameters symptom status (stacked bars on the left) and treatment satisfaction (stacked bars on the right) at the end of follow‐up after catheter ablation are displayed. Antiarrhythmic drugs comprised Vaughan Williams Class I and III. Betablocker therapy was part of the treatment in both groups. *p* < .05 was considered statistically significant, there were no significant differences between groups. AAD, antiarrhythmic drugs, *p*, *p*‐Value, %, per cent

After catheter ablation for pAF 81.5% (785/963) of the AAD group versus 84.2% (837/994) of the no AAD group reported either no remaining symptoms or reduced symptoms (*p* = .11; see Figure 3A).

Patients treated for persAF reported no remaining symptoms or reduced symptoms in 75.9% (438/577, AAD group) versus 76.6% (341/445, no AAD group, *p* = .79; see Figure 3B).

The multivariable regression analysis of patients with improvement of symptoms showed no significant differences between groups in pAF and persAF patients, respectively (see Figure 2).

#### 3.5.2 Patients’ treatment satisfaction

Patients with pAF discharged with AAD were significantly less often fully satisfied with their treatment (treatment “successful” in 61.1% (149/244) vs. 70.6% (207/293, *p* = .019; see Figure 3A)). Patients reported “partial success” in 22.5% (55/244) versus 17.7% (52/293, *p* = .17) and “no success” in 16.4% (40/244) versus 10.6% (31/293, *p* = .048). These results were consistent after adjustment for differences in the baseline parameters (OR with 95% CI for patients not fully satisfied: 1.76 [1.20–2.58]; Figure 2).

In patients with persAF no significant differences in treatment satisfaction were reported (full success: 58.8% (94/160) versus 63.7% (72/113), *p* = .41; partial success 22.5% (36/160) versus 21.2% (24/113), *p* = .80; no success 18.1% (29/160) versus 15.0% (17/113), *p* = .50; adjusted OR 1.24, 95% CI [0.74–2.08]; see Figure 3B).

Data on treatment satisfaction were not available for all patients as this parameter was included into the trial during a protocol amendment.

## 4 DISCUSSION

The main findings of this study are:

1. In patients with pAF the recurrence and rehospitalization rates after catheter ablation did not differ between individuals discharged with or without postinterventional AAD in the adjusted analysis. However, the reablation rate was higher in patients discharged with AAD.
2. Patients with pAF discharged with AAD rated the treatment as “not successful” more often than patients discharged without AAD.
3. In patients with persAF the rates of recurrences, rehospitalizations and reablations did not differ between individuals discharged with or without AAD in the adjusted analysis.
4. No matter if discharged with or without postinterventional AAD, the rates of cardiovascular events did not differ in patients with pAF and persAF during long‐term follow‐up.

### 4.1 Recurrence and reablation rates

For many AF patients, the freedom from AAD is one of the desired accomplishments of successful catheter ablation. Still, clinicians sometimes hesitate to discontinue AAD as they are worried about recurrences, which are a common observation during the treatment of AF, even after catheter ablation. However, data on the optimal treatment strategy regarding AAD after catheter ablation are still sparse and partly contradictory.^9^ Besides a permanent PVI, a longer‐lasting treatment with AAD might be a strategy to less recurrences during long‐term observance, as shown by the POWDER AF study.^10^ This approach is especially interesting for patients with long‐standing persAF, who have limited long‐term success by PVI alone, and yet no proven better ablation approach.^11^, ^12^ Our analysis contributes insights from a large, prospective multicenter registry. Observing the 12‐months period after ablation we found no reduction in recurrences and rehospitalizations in patients with pAF and persAF when discharged with AAD. Thus, our data stand in line with experiences from the EAST‐AF, AMIO‐CAT, and also the 5A trial, which investigated postinterventional AAD.^7^, ^8^, ^13^, ^14^ Despite less recurrences during the limited “blanking period,” none of those trials showed an overall reduction of recurrences during long‐term follow‐up. A possible reason might be, that besides the temporary postinterventional inflammatory triggers of AF,^15^ gaps in the circular PVI lesion or unsuccessful ablation of triggers unrelated to pulmonary veins are potential causes of long‐term recurrences.^16^ On the contrary, the POWDER AF study did report less recurrences during long‐term follow‐up for pAF patients with continued AAD; however, it followed a different approach, including only individuals without recurrences during the “blanking period.”^10^ The above‐mentioned considerations of long‐term AAD might not fit for all AF patients. While reablation rates in persAF patients in the present study were not reduced by postinterventional AAD, pAF patients had a 1.3 times higher rate of reablations when AAD was prescribed at discharge. This effect was observed, regardless of similar recurrence rates in patients discharged with or without AAD. Hypothetically, while the recurrences themselves were tolerable for the patients, the perspective of a prolongation of AAD, which was probably suggested as an alternative option to reablation in patients discharged with AAD, motivated the relatively healthy pAF patients to undergo another ablation procedure.

### 4.2 Patient satisfaction with treatment

Patient reported outcomes are an important part of integrated AF management as the patients’ experience of arrhythmia and treatment burden are often subjective and not always represented in the usual clinical endpoints.^9^, ^17^ In our present analysis, pAF patients discharged without AAD had a significantly higher level of satisfaction with treatment than all other groups (pAF discharged with AAD, persAF discharged with/without AAD). A detailed analysis by Brachmann et al.,^18^ including all patients with supraventricular arrhythmias from the German Ablation Registry showed that treatment satisfaction is often influenced by arrhythmia recurrences. At least in patients with pAF this seems not to be the case, as recurrences did not differ significantly between groups. However, pAF patients discharged with AAD had a significantly higher rate of AF reablations as compared to patients without AAD use, which might influence treatment satisfaction. A study by Kany et al. found that patient satisfaction was similar after primary and reablation procedures.^19^ This leads to the conclusion that at least in the group of patients with pAF postinterventional AAD might reduce treatment satisfaction. Patients with persAF are possibly more used to medication use (due to a higher number of comorbidities) and were not so much bothered by additional drug treatment.

### 4.3 Adverse events during follow‐up

Data from the EAST‐AFNET4 trial revealed that an early rhythm control therapy regime can reduce the likelihood of cardiovascular events in patients with AF.^20^ It is still unclear whether a combined treatment approach using catheter ablation and AAD reduces event rates more than one of the options alone. In our analysis rates of cardiovascular events were low and the adjusted analysis showed no significant differences in the occurrence of the combined endpoint “MACCE” between patients discharged with or without AAD. This was observed in both patients with pAF and persAF.

### 4.4 Limitations

A limiting factor of this analysis might be the varying duration of the AAD intake, as some patients of the AAD group discontinued AAD, whereas patients without AAD started antiarrhythmic drugs during follow‐up. While the number of patients on medication at the end of follow‐up is shown above, the documentation of the exact duration of intake of antiarrhythmic medication has not been part of the study protocol. The ablation procedures included into this analysis were performed several years ago and technological advances with impact on ablation outcome have been made since then. Prescription of antiarrhythmic drugs was at will of the study center, and confounding is possible despite having corrected the baseline parameters in the regression analysis. Although derived from a large prospective multicenter registry, our study should be considered hypothesis generating due to the retrospective character of the analysis. Randomized studies are needed to confirm our results and further clarify the postinterventional use of AAD.

## 5 CONCLUSION

Patients discharged with AAD after AF catheter ablation had similar rates of recurrences, rehospitalizations and cardiovascular events during follow‐up as patients discharged without AAD. However, in pAF patients AAD at discharge was associated with reduced treatment satisfaction and a higher reablation rate. Therefore, postinterventional prescription of AAD has to be carefully considered, especially after ablation of pAF.

## DISCLOSURE

RS reports no conflicts of interest. AM reports travel grants and speaker fees from Medtronic and Biosense Webster. KK reports research contracts/grants from Biosense Webster, Medtronic, Abbott Vascular, and Boston Scientific and consulting fees from Abbott Vascular. SW reports speakers bureau/study funding from Abbott and Boston Scientific; speakers bureau from Boehringer Ingelheim, Bristol Myers Squibb, Bayer Vital, and Daiichi‐Sankyo. EH received compensation for participation in clinical research trials outside the submitted work from Abbott, Bayer, Biotronik, Boehringer Ingelheim, Edwards, Elixier, Medtronic and Stentys. DT received lecture fees/honoraria from Bayer Vital, Boehringer Ingelheim Pharma, Bristol‐Myers Squibb, Daiichi Sankyo, Medtronic, Pfizer Pharma, Sanofi‐Aventis, St. Jude Medical and ZOLL CMS. LE received research support from several drug and device companies active in the field of electrophysiology and received honoraria from several such companies in the past. JS and MH: the long‐term follow‐up and prior publications were partially supported by unrestricted grants from Medtronic, Biosense Webster, and Biotronik. AR reports travel grants and speaker fees from Biosense Webster, Hansen Medical, Medtronic, EPSolutions and St. Jude Medical as well as speaker fees from Boehringer Ingelheim and consulting fees from Medtronic.

## AUTHORS’ CONTRIBUTIONS

RS and AR take responsibility for all aspects of the reliability and freedom from bias of the data presented and their discussed interpretation. RS and AR analyzed and interpreted the data and wrote the manuscript. MH and JS contributed to the acquisition of the data, design of the study, analysis and interpretation of the data and critically revised the manuscript. AM, KK, DA, SW, EH, TD, LE, and JB contributed to the acquisition and interpretation of the data and critically revised the manuscript.

## ETHICS APPROVAL/CONSENT TO PARTICIPATE

The study has been approved by the local ethics committee and has therefore been performed in accordance with the ethical standards laid down in the 1964 Declaration of Helsinki and its later amendments. All individual participants included into the study gave written informed consent. Patients signed informed consent regarding publishing their data.

## Supporting information

Supplementary Material

## Data Availability

The underlying data are available upon reasonable request.
