# Supplementary material for: Antiarrhythmic drug therapy after catheter ablation for atrial fibrillation—Insights from the German Ablation Registry
Source: Pharmacol Res Perspect. 2021 Oct 19;9(6):e00880. doi: 10.1002/prp2.880 (PMC8525107; doi:10.1002/prp2.880)
Supplement: Supplementary file 1 — Supplementary Material [file PRP2-9-e00880-s001.zip › prp2880-sup-0001-Supplements.docx]

# Supplemental Material

## Supplemental Figure 1 12-Months Outcome Analysis (Univariate Analysis).

## A Patients with Paroxysmal Atrial Fibrillation

##

## B Patients with Persistent Atrial Fibrillation

##

## The outcome parameters recurrence, reablation and rehospitalization during follow-up after catheter ablation for persistent atrial fibrillation are displayed. The blue bars indicate patients that were discharged with antiarrhythmic drug therapy after catheter ablation, the brown bars indicate patients that were discharged without antiarrhythmic drug therapy. Antiarrhythmic drugs comprised Vaughan Williams Class I and III. Betablocker therapy was allowed in both groups. p<0.05 was considered statistically significant. Significant differences in results are marked with capped lines.

Abbreviations: AAD indicates antiarrhythmic drugs; p, p-Value; %, percent.

## Supplemental Figure 2 12-Months Analysis of Patient Safety (Unadjusted Analysis).

## A Patients with Paroxysmal Atrial Fibrillation

##

## B Patients with Persistent Atrial Fibrillation (unadjusted Analysis)

##

## The outcome parameters Mortality, MACE (death, myocardial infarction), MACCE (death, myocardial infarction, ischemic stroke), Quadruple Safety Endpoint (death, myocardial infarction, ischemic stroke, major bleeding) during follow-up after catheter ablation for paroxysmal atrial fibrillation were estimated using the Kaplan Meier Method. Furthermore, incident rates of severe complications (myocardial infarction, ischemic stroke, major bleeding) and moderate complications (syncope, transient ischemic attack, systemic embolism, pulmonary embolism, deep vein thrombosis, cardiopulmonary resuscitation, moderate bleeding, percutaneous coronary intervention, groin related symptoms) during follow-up are displayed. The blue bars indicate patients that were discharged with antiarrhythmic drug therapy after catheter ablation, the purple bars indicate patients that were discharged without antiarrhythmic drug therapy. Antiarrhythmic drugs comprised Vaughan Williams Class I and III. Betablocker therapy was allowed in both groups.

Abbreviations: AAD indicates antiarrhythmic drugs; KM, Kaplan Meier; MACE, major adverse cardiac event; MACCE, major adverse cardiac and cerebrovascular event; %, percent.

## Supplemental Table 1a Adverse Events during Follow-up (Patients with Paroxysmal Atrial Fibrillation)

|  | **AAD (n=1051)** | **No AAD (n=1087)** | **p-Value** |
| --- | --- | --- | --- |
| Severe Complications | 1.5% | 1.2% | 0.49 |
| Myocardial Infarction | 0.3% | 0.0% | 0.077 |
| Ischemic Stroke | 0.4% | 0.3% | 0.66 |
| Major Bleeding | 0.8% | 0.9% | 0.88 |
| Moderate Complications | 9.1% | 7.4% | 0.22 |
| Syncope | 0.4% | 0.2% | 0.38 |
| Transient Ischemic Attack | 0.6% | 0.4% | 0.48 |
| Systemic Embolism | 0.1% | 0.0% | 0.31 |
| Pulmonary Embolism | 0.1% | 0.1% | 0.98 |
| Deep Vein Thrombosis | 0.2% | 0.1% | 0.53 |
| Cardiopulmonary Resuscitation | 0.2% | 0.0% | 0.15 |
| Moderate Bleeding | 1.8% | 1.3% | 0.39 |
| Coronary revascularization | 0.8% | 1.6% | 0.12 |
| Groin Access Related Symptoms | 4.6% | 3.5% | 0.21 |

## Supplemental Table 1b Adverse Events during Follow-up (Patients with Persistent Atrial Fibrillation)

|  | **AAD (n=641)** | **No AAD (n=496)** | **p-Value** |
| --- | --- | --- | --- |
| Severe Complications | 2.1% | 2.7% | 0.52 |
| Myocardial Infarction | 0.2% | 0.4% | 0.42 |
| Ischemic Stroke | 0.7% | 0.9% | 0.71 |
| Major Bleeding | 1.2% | 1.6% | 0.62 |
| Moderate Complications | 6.6% | 8.2% | 0.37 |
| Syncope | 0.5% | 0.5% | 0.87 |
| Transient Ischemic Attack | 0.0% | 0.7% | 0.049 |
| Systemic Embolism | 0.2% | 0.0% | 0.38 |
| Pulmonary Embolism | 0.2% | 0.0% | 0.38 |
| Deep Vein Thrombosis | 0.2% | 0.5% | 0.42 |
| Cardiopulmonary Resuscitation | 0.3% | 0.2% | 0.72 |
| Moderate Bleeding | 0.9% | 0.9% | 0.97 |
| Coronary revascularization | 1.2% | 2.0% | 0.30 |
| Groin Access Related Symptoms | 3.0% | 3.9% | 0.44 |

Values are presented as percent (%) of available data sets. p<0.05 is considered statistically significant.

Abbreviations: AAD indicates antiarrhythmic drugs.
