# Supplementary material for: Antiarrhythmic drug therapy after catheter ablation for atrial fibrillation—Insights from the German Ablation Registry
Source: Pharmacol Res Perspect. 2021 Oct 19;9(6):e00880. doi: 10.1002/prp2.880 (PMC8525107; doi:10.1002/prp2.880)
Supplement: Supplementary file 1 — Supplementary Material [file PRP2-9-e00880-s001.zip › prp2880-sup-0002-Supplement_Questionnaire.doc]

Zentrum:	|__|__|__|__|	Patient-ID:	|__|__|__|

Ablation:	|__|__||__|__||__|__|

Datum der Nachbeobachtung	|__|__| * __|__| * __|__|__|__|

Status des Patienten	 lebt  verstorben am: |__|__| * __|__| * __|__|__|__|

 Kardiale Todesursache

 Nicht-kardiale Todesursache

 Plötzlicher Tod

 Unbekannte Todesursache

Sozialstatus	❑ berentet	❑ in Arbeit	❑sonstiges/unbekannt

Anginabeschwerden:	 nein	 ja	 unbekannt

NYHA	❑ keine/NYHA1 ❑ NYHA2 ❑ NYHA3 ❑ NYHA4	 unbekannt  n.b.

Rezidiv	❑ nein	❑ ja, am|__|__| * __|__| * __|__|__|__ ❑ unbekannt

❑ dokumentiert	Konsequenz:______________

Erneute Ablation	 nein	 ja, am |__|__| * __|__| * __|__|__|__|  unbekannt

Ohnmacht/Bewusstlosigkeit	 nein	 ja, am |__|__| * __|__| * __|__|__|__|  unbekannt

Implantation	❑ nein	❑ unbekannt

❑ ja	❑ SM ❑ ICD ❑ CRT am: |__|__| * __|__| * __|__|__|__| PV-Stenose

(Atemnot,Bluthusten)	❑ nein	❑ unbekannt	❑ ja	__|__| %

Intervention:❑ nein	❑ ja	❑ unbekannt

Phrenicus-parese	 nein	 ja	 unbekannt

(Leistungsknick,Atemnot)

Atrio-ösophageale Fistel	 nein	 ja	 unbekannt

(Schluckstörungen,Magen/Darmspiegelung, Behandlung mit Antibiotika)

Leistenprobleme	 nein	 ja	 unbekannt

Myokardinfarkt	 nein	 ja, am |__|__| * __|__| * __|__|__|__|  unbekannt

Stroke	 nein	 ja, am |__|__| * __|__| * __|__|__|__|  unbekannt

TIA	 nein	 ja, am |__|__| * __|__| * __|__|__|__|  unbekannt

Systemische Embolie	 nein	 ja, am |__|__| * __|__| * __|__|__|__|  unbekannt

Lungenembolie	 nein	 ja, am |__|__| * __|__| * __|__|__|__|  unbekannt

Venenthrombose (Bein/Becken)  nein  ja, am |__|__| * __|__| * __|__|__|__|  unbekannt

Reanimation	 nein	 ja, am |__|__| * __|__| * __|__|__|__|  unbekannt

Schwere Blutung	 nein	 ja, am |__|__| * __|__| * __|__|__|__|  unbekannt

(mit ärztlicher Intervention)

Moderate Blutung	 nein  unbekannt	 ja, am |__|__| * __|__| * __|__|__|__|

(Absetzen/Dosisreduktion der antithrombotischen Therapie)

Revaskularisation	 nein	 ja, am |__|__| * __|__| * __|__|__|__|  unbekannt

 PCI	 ACB	 unbekannt

Symptomatik	 keine	 gebessert	 unbekannt

 unverändert	 schlechter

Rehospitalisierung	 nein	 ja am |__|__| * __|__|	 unbekannt

Anzahl	|__|__|	Geamtdauer in Tagen	|__|__|

Ursache(n):

Medikamenteneinnahme:

Adressdaten dürfen für evtl. spätere Befragungen gespeichert und verwendet werden

 nein	ja

_______________

Unterschrift
